# Supplementary material for: Content-rich biological network constructed by mining PubMed abstracts
Source: BMC Bioinformatics. 2004 Oct 8;5:147. doi: 10.1186/1471-2105-5-147 (PMC528731; doi:10.1186/1471-2105-5-147)
Supplement: Additional File 2 — The original results of the above study (non-essential files are deleted to keep the file size under the limit set by BMC bioinformatics). [file 1471-2105-5-147-S2.bz2 › chilibotAdditionalFile2/dip10/747ID9150216E18129/html/EXBB_TONB.html]

 


 **EXBB** and **TONB** 
  
Found 83 abstracts in PubMed, retrieved 10.  
 

 What does Google say? 
 PDF only 
| .edu only 

---

**Interactive relationship** (e.g. stimulation, inhibition, etc)

**Neutral relationship**- **ExbB**  and ExbD proteins are part of the  **TonB**  dependent energy transduction system and are encoded by the exb operon in Escherichia coli.  Ref: 12193634 J Bacteriol, 2002
- Both  **ExbB**  and ExbD were also required for association of  **TonB**  with the cytoplasmic membrane.  Ref: 12193634 J Bacteriol, 2002
- Specifically, both  **ExbB**  and ExbD were found to be required for  **TonB**  to conformationally respond to proton motive force.  Ref: 12193634 J Bacteriol, 2002
- The aim of this review is to describe an in vivo assay of the interactions taking place in the Tol Pal or  **TonB**   **ExbB**  ExbD envelope complexes in the periplasm of Escherichia coli and between them and colicins or g3p protein of filamentous bacteriophages.  Ref: 12423784 Biochimie< MedlineTA>Biochimie, 2002

**Non-interactive relationship** (e.g. studied together, co-existance, homology, etc.)

- **TonB** , the energy transducer, appears to go through a cycle during energy transduction, with the absence of both  **ExbB**  and ExbD creating blocks at two points i in the inability of  **TonB**  to respond to the cytoplasmic membrane proton motive force and ii in the conversion of  **TonB**  from a high affinity outer membrane association to a high affinity cytoplasmic membrane association.  Ref: 12193634 J Bacteriol, 2002
- Reverse transcription RT PCR, using RNA from organisms grown under iron replete and iron restricted conditions, revealed that  **tonB** ,  **exbB** , exbD, tbpB and tbpA are transcribed as a single unit with expression being up regulated in response to iron restriction.  Ref: 12742718 Vet Microbiol, 2003
- PCR approaches allowed the amplification, cloning and sequencing of A. suis  **tonB** ,  **exbB** , exbD, tbpB and tbpA homologues.  Ref: 12742718 Vet Microbiol, 2003
- A complex of three membrane spanning proteins  **TonB** ,  **ExbB**  and ExbD couples the chemiosmotic potential of the cytoplasmic membrane with siderophore uptake across the outer membrane.  Ref: 12409204 Biochim Biophys Acta, 2002
- The Ton system consisting of the proteins  **TonB** ,  **ExbB**  and ExbD is required for colicin and microcin import.  Ref: 12423780 Biochimie< MedlineTA>Biochimie, 2002
- This gene is clustered with three other genes,  **tonB**   **exbB**  exbD1 exbD2.  Ref: 12646254 Biochem Biophys Res Commun, 2003
- **ExbB**  and ExbD do not function independently in  **TonB**  dependent energy transduction.  Ref: 12193634 J Bacteriol, 2002
- Results of insertional mutations, DNA and protein sequence analyses, phage sensitivity tests, transfection tests, complementation tests, and phage adsorption assays together with the cellular location of the proteins indicate that  **TonB** ,  **ExbB** , and ExbD1 are essential for penetration of phage phi L7.  Ref: 12646254 Biochem Biophys Res Commun, 2003
- In gram negative organisms, high affinity transport of iron substrates requires energy transduction to specific outer membrane receptors by the  **TonB**   **ExbB**  ExbD complex.  Ref: 12562789 J Bacteriol, 2003
- Both full length  **TonB**  and truncated  **TonB**  consisting of the amino terminal two thirds achieved identical conformations in the cytoplasmic membrane, as determined by their abilities to cross link to the cytoplasmic membrane protein  **ExbB**  and their abilities to respond conformationally to the presence or absence of proton motive force.  Ref: 12823822 Mol Microbiol, 2003
- The  **exbB** , exbD and  **tonB**  genes of the Pasteurella multocida animal pathogen have been cloned by complementation of an Escherichia coli  **tonB**  mutant.  Ref: 12044675 FEMS Microbiol Lett, 2002
- Pasteurella multocida  **exbB** , exbD and  **tonB**  genes are physically linked but independently transcribed.  Ref: 12044675 FEMS Microbiol Lett, 2002
- The energy is transduced to FecA by the inner membrane complex,  **TonB** ,  **ExbB** , and ExbD.  Ref: 12618451 J Bacteriol, 2003
